# Direct interaction of avermectin with epidermal growth factor receptor mediates the penetration resistance in *Drosophila* larvae

**DOI:** 10.1098/rsob.150231

**Published:** 2016-04-13

**Authors:** Li-Ping Chen, Pan Wang, Ying-Jian Sun, Yi-Jun Wu

**Affiliations:** 1 Laboratory of Molecular Toxicology, State Key Laboratory of Integrated Management of Pest Insects and Rodents, Institute of Zoology, Chinese Academy of Sciences, Beijing 100101, People's Republic of China; 2 Department of Veterinary Medicine and Animal Science, Beijing Agriculture College, Beijing 102206, People's Republic of China; 3 University of Chinese Academy of Sciences, Beijing 100049, People's Republic of China

**Keywords:** avermectin, penetration resistance, *DmeCHS1/2*, EGFR, *Drosophila melanogaster* larvae

## Abstract

With the widespread use of avermectins (AVMs) for managing parasitic and agricultural pests, the resistance of worms and insects to AVMs has emerged as a serious threat to human health and agriculture worldwide. The reduced penetration of AVMs is one of the main reasons for the development of the resistance to the chemicals. However, the detailed molecular mechanisms remain elusive. Here, we use the larvae of *Drosophila melanogaster* as the model organism to explore the molecular mechanisms underlying the development of penetration resistance to AVMs. We clearly show that the chitin layer is thickened and the efflux transporter P-glycoprotein (P-gp) is overexpressed in the AVM-resistant larvae epidermis. We reveal that the activation of the transcription factor Relish by the over-activated epidermal growth factor receptor (EGFR)/AKT/ERK pathway induces the overexpression of the chitin synthases *DmeCHS1/2* and P-gp in the resistant larvae. Interestingly, we discover for the first time, to the best of our knowledge, that AVM directly interacts with EGFR and leads to the activation of the EGFR/AKT/ERK pathway, which activates the transcription factor Relish and induces the overexpression of *DmeCHS1/2* and P-gp. These findings provide new insights into the molecular mechanisms underlying the development of penetration resistance to drugs.

## 1. Introduction

Avermectins (AVMs), which are macrocyclic lactones initially extracted from *Streptomyces avermitilis* [1,2], are highly effective biological anthelmintics and are widely used for the management of agricultural and parasitic infections. AVMs, used as insecticides, acaricides and nematicides, can eradicate 80 kinds of worms and insects, such as nematodes, mites and lice [3]. With the widespread use of AVMs, the resistance to AVMs in worms and insects as well as the toxic effects of AVMs on the parasite carriers such as humans and animals are becoming increasingly serious [4–7], and are detrimental to human health and agriculture.

Numerous studies have revealed two important mechanisms for the development of resistance to AVMs, i.e. target insensitivity and increased metabolism of AVMs [8,9]. AVMs mainly bind to the glutamate-gated chloride channel and *γ*-aminobutyric acid-gated chloride channel and result in the release of chloride in insects and worms [10,11]. This binding is essentially irreversible, leading to a very long-lasting hyperpolarization or depolarization of the neurons or muscle cells, subsequently blocking their further function [12,13]. Gene mutations or downregulation of chloride channels can result in target insensitivity, which confers the development of target resistance in animals to AVMs [8,14]. Meanwhile, AVMs can be metabolized by oxidation and hydroxylation, which increase the polarity of AVMs [15–17]. Elevated oxidation activity of enzymes such as P450 and glutathione *S*-transferase can result in metabolism resistance to AVMs in insects [9,18].

Besides these two mechanisms, reduced penetration of insecticides in insects is another mechanism underlying the development of the resistance to insecticides [19–23]. Because AVMs mainly kill worms and insect larvae via contact [23,24], reduction in epidermal permeability is potentially related to the AVM resistance in worms and insects. However, the molecular mechanisms underlying the reduction of epidermal permeability in AVM-resistant worms and insect larvae are still unclear.

**Figure 1.**
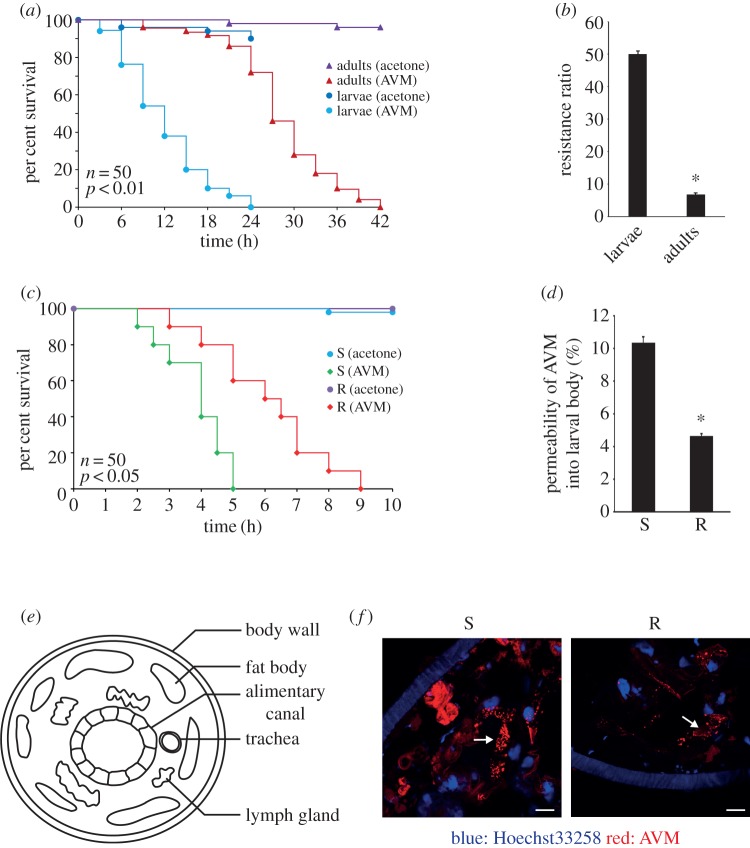
Penetration resistance is observed in avermectin-resistant *Drosophila* larvae. (*a*) Percentage of surviving avermectin-susceptible larvae and adults spotted with 0.25 µl of acetone or 1 mM avermectin (AVM). (*b*) Resistance ratio of larvae and adults to AVM. (*c*) Percentage of surviving larvae spotted with 0.25 µl of 100 mM AVM. (*d*) Larvae were spotted with 0.25 µl of 10 mM AVM and then cultured in regular medium for 2 h. Permeability of AVM was then detected by HPLC analysis. (*e*) Diagram of transverse section of a larva. (*f*) Immunofluorescence staining of AVM in the fat body tissue of AVM-susceptible strain (S) and AVM-resistant strain (R) larvae which were treated with 1 mM AVM for 3 h. Blue: nuclei; red: AVM (scale bar, 20 µm). Data were expressed as mean ± s.e.m., *p < 0.01, compared with the corresponding group, *n* = 3.

*Drosophila melanogaster* is a widely used model organism for insects. In addition, the *Drosophila* larvae are similar to nematode worms in numerous biological processes [25–27]. Because AVMs are a kind of larvicidal insecticide [28,29], we use *Drosophila* larvae as the model organism in this study for both worms and insects to explore the mechanisms underlying the development of penetration resistance to AVM. In addition, although the *Drosophila* adults were used in our previous study [30], we found that the larvae were more sensitive to AVM than the adults (figure 1). Thus, we switched to using larvae of *D. melanogaster* as the model organism in this study. Here, we identify a clear molecular mechanism underlying the penetration resistance to AVM in *Drosophila*.

## 2. Results

### 2.1. The penetration resistance shown in the avermectins-resistant larvae

We first sought to compare the sensitivities of AVM-susceptible strain *Drosophila* larvae and adults with AVM. We found that the larvae died significantly faster than the adults when they were exposed to the same doses of AVM (figure 1*a*). Meanwhile, we found that the resistance ratio of the larvae was drastically higher than that of the adults (figure 1*b* and electronic supplementary material, figure S1). These findings indicate that the larvae are more sensitive to AVM than adults. As a result, *Drosophila* larvae were chosen as the model organism in this study. Indeed, when exposed to the same concentrations of AVM, the AVM-susceptible larvae (S larvae) died significantly faster than the AVM-resistant larvae (R larvae; figure 1*c*), which suggests that R larvae are resistant to AVM toxicity.

High-performance liquid chromatography (HPLC) analysis results showed that the permeability of AVM in the larval body of the resistant strain was only half of that in the susceptible strain (figure 1*d*). Previous studies have shown that AVMs mainly exist in the original form in the body of vertebrate animals [31,32], and metabolites of AVM in *Drosophila* have not been reported as yet. Thus, in this study, only the original form of AVM was detected by HPLC, partly owing to the short treatment time. Immunofluorescence analysis also showed that the amount of AVM accumulated in the fat body of R larvae was lower than that in S larvae, after the larvae were treated with the same concentration of AVM for the same time (figure 1*e,f*). These results indicate that AVM can penetrate more easily into S larvae, which leads to the faster death of S larvae. Thus, the reduced penetration of AVM into R larvae results in their resistance to AVM.

### 2.2. Upregulation of P-gp and chitin synthases in the avermectins-resistant larvae

**Figure 2.**
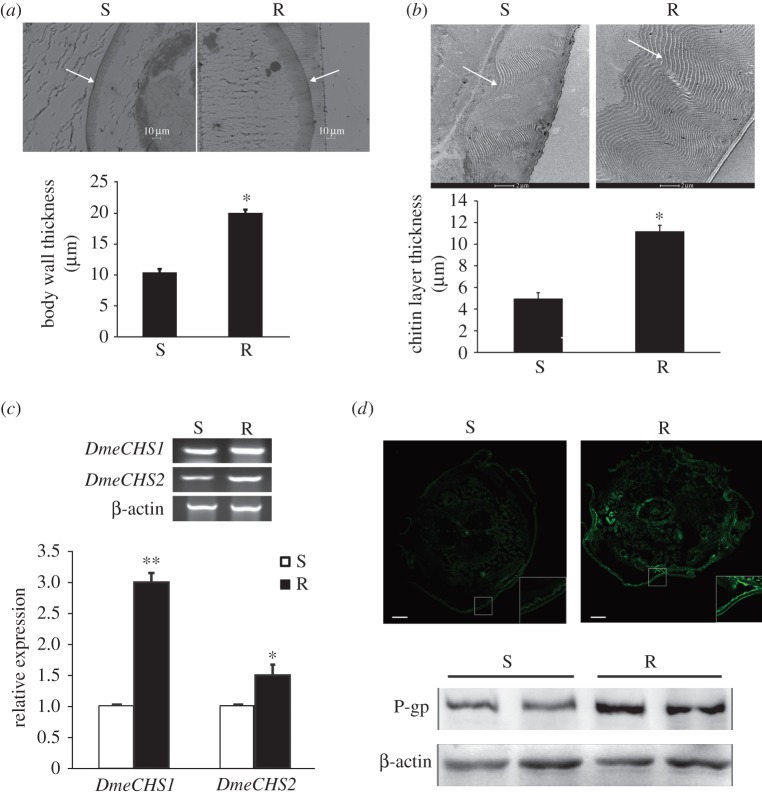
P-gp and chitin synthases are upregulated in avermectin-resistant *Drosophila* larvae. (*a,b*) Thickness of larvae body wall (*a*) and chitin layer (*b*) was determined by microscopy. Microscopic pictures showed the cross section of the body wall or the chitin layer in cuticle. (*c*) The mRNA levels of chitin synthases *DmeCHS1* and *DmeCHS2* in larvae detected by RT-PCR and q-PCR. (*d*) The protein level of P-gp was detected by immunofluorescence and western blotting analysis. Green: P-gp (scale bar, 100 µm). Data were expressed as mean ± s.e.m., *p < 0.05, **p < 0.01, compared with the corresponding avermectin-susceptible (S) larvae group, *n* = 3.

Epidermis is the first and most important barrier that restricts the diffusion of chemicals including AVMs into the animal body. To investigate whether the penetration resistance to AVM is related to the epidermis, the structure of S and R larvae body wall was examined with the optical microscope. The semi-thin slice results showed that R larvae body wall was about twice as thick as S larvae body wall (figure 2*a*). In addition, electron microscopy showed that the chitin layer in R larvae was approximately twice as thick as that in S larvae (figure 2*b*). The alimentary canal is another important barrier that restricts the diffusion of AVM. The peritrophic matrix (PM) is composed of chitin and glycoproteins and lines the insect intestinal lumen, and the PM can protect the midgut epithelium from mechanical damage, pathogens and toxins [31]. The electron microscopy results showed that the thickness of the PM of S and R larvae was the same (electronic supplementary material, figure S2). These results indicate that a thickened chitin layer in the epidermis is probably one of the main reasons for the reduced penetration of AVM in R larvae.

In *D. melanogaster* larvae, there are two genes for chitin synthases, i.e. *DmeCHS1* and *DmeCHS2*, which belong to the insect *CHS-A* and *CHS-B* gene families, respectively [33,34]. *DmeCHS1* and *DmeCHS2* are mainly expressed in the epidermis and other tissues, respectively. The RT-PCR and q-PCR analysis results showed that mRNA levels of the two chitin synthases, *DmeCHS1* and *DmeCHS2*, in R larvae were higher than those in S larvae (figure 2*c*), especially for *DmeCHS1*. These results indicate that the overexpression of chitin synthases probably contributes to the thickened chitin layer in R larvae.

**Figure 3.**
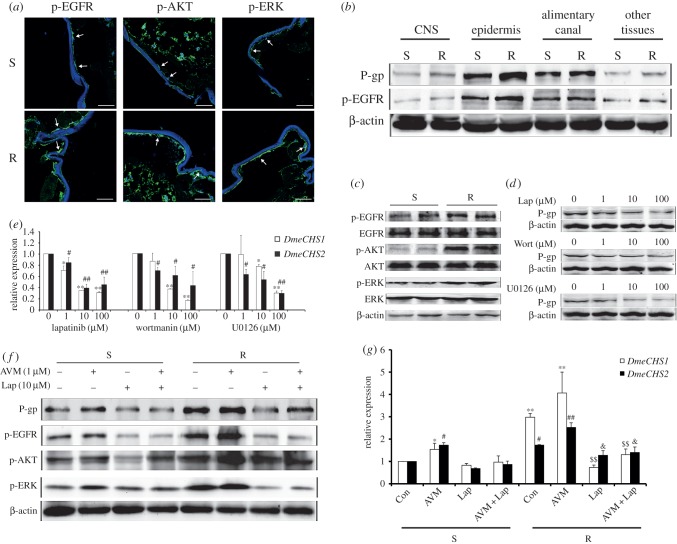
Expression of P-gp and *DmeCHS1/2* is regulated by EGFR/AKT/ERK signalling pathway. (*a*) The protein levels of p-EGFR, p-AKT and p-ERK in the epidermal cells of the body wall of the *Drosophila* larvae were detected by immunofluorescence. Blue: nuclei stained by Hoechst33258; green: target proteins (scale bar, 50 µm). (*b*) The expression levels of P-gp and p-EGFR in different tissues of the larvae. (*c*) The expression levels of p-EGFR, p-AKT, p-ERK and their three respective total proteins were detected by western blotting analysis. (*d,e*) Avermectin-susceptible (S) larvae were treated with different concentrations of lapatinib (Lap), wortmanin (Wort), or U0126, respectively, for 48 h. The protein levels of P-gp were detected by western blotting analysis (*d*), and the mRNA levels of *DmeCHS1* and *DmeCHS2* were detected by qPCR analysis with β-actin as the internal control (*e*). (*f,g*) The larvae were treated with avermectin (AVM) and lapatinib for 48 h. The protein levels (*f*) of P-gp, p-EGFR, p-AKT and p-ERK and the mRNA levels (*g*) of *DmeCHS1* and *DmeCHS2* in larvae treated with AVM and lapatinib were determined by western blotting analysis and qPCR analysis with β-actin as the internal control, respectively. Data were expressed as mean ± s.e.m., *p < 0.05, **p < 0.01, ^#^*p* < 0.05, ^##^*p* < 0.01, compared with the corresponding S control group; ^$^*p* < 0.05, ^$$^*p* < 0.01, ^&^*p* < 0.05, ^&&^*p* < 0.01, compared with the corresponding avermectin-resistant (R) control group; *n* = 3.

In the epidermal cells of animals, P-glycoprotein (P-gp) is a key factor that regulates the penetration of AVM, because it can transport AVM out of cells [35]. P-gp is one of the ATP-dependent membrane transport proteins, and P-gp is mainly expressed in barrier tissues, such as epidermis, digestive epithelium and the blood–brain barrier (BBB). Western blotting and immunofluorescence analysis results showed that P-gp was upregulated in R larvae, particularly in the body wall (figures 2*d* and 3*b*). In conclusion, the higher expression of P-gp and chitin synthases in resistant larvae probably leads to the reduction of AVM permeability in R larvae.

### 2.3. Activation of EGFR/AKT/ERK pathway upregulates the expression of P-gp and *DmeCHS1/2*

Chitin synthases are upregulated during development and epidermal wound-healing [36], and the tyrosine kinase receptor epidermal growth factor receptor (EGFR) signalling pathway is also activated in epidermal wound-healing models [37]. Thus, the EGFR pathway may regulate chitin synthases in insecticide resistance. In addition, the expression of P-gp was found to be regulated by the EGFR/AKT pathway in *Drosophila* adults [30]. In order to investigate whether the EGFR/AKT/ERK pathway is activated in *Drosophila* larvae, immunofluorescence analysis and western blotting analysis were used.

We found that the EGFR/AKT/ERK pathway was activated in epidermal cells of the resistant strain (figure 3*a,c*). In addition, P-gp and p-EGFR were mainly expressed in the epidermis of larvae and upregulated in the epidermis of R larvae (figure 3*b*). P-gp expression in the susceptible strain was inhibited after the treatment with the EGFR phosphorylation inhibitor lapatinib, AKT phosphorylation inhibitor wortmanin and ERK phosphorylation inhibitor U0126 (figure 3*d*). Furthermore, these inhibitors reduced the mRNA levels of *DmeCHS1* and *DmeCHS2* in S larvae (figure 3*e*). These results indicate that the EGFR/AKT/ERK pathway regulates the expression of P-gp and chitin synthases *DmeCHS1* and *DmeCHS2*.

To demonstrate whether the activation of the EGFR/AKT/ERK pathway indeed induces the overexpression of the proteins P-gp, *DmeCHS1* and *DmeCHS2* in the resistant strain, the larvae of the two strains were treated with AVM and lapatinib. Western blotting analysis and q-PCR assay results showed that AVM enhanced P-gp expression and *DmeCHS1/2* mRNA levels in both the two strains of larvae (figure 3*f,g*). Moreover, lapatinib inhibited the basal level of P-gp expression and *DmeCHS1/2* mRNA levels in both S and R larvae, and further inhibited the AVM-induced overexpression of P-gp and *DmeCHS1/2*. Notably, the expression of P-gp and *DmeCHS1/2* in R larvae was suppressed by lapatinib to levels comparable with those in S larvae (figure 3*f,g*). In addition, wortmanin and U0126 had similar effects in regulating P-gp expression compared with lapatinib. Both wortmanin and U0126 reduced P-gp and *DmeCHS1/2* mRNA levels in S and R larvae and suppressed the overexpression of P-gp and *DmeCHS1/2* in R larvae (electronic supplementary material, figure S3). Altogether, these findings suggest that the overexpression of P-gp and chitin synthases *DmeCHS1/2* in R larvae is induced by the activation of the EGFR/AKT/ERK pathway.

### 2.4. Activation of Relish mediates the overexpression of P-gp and *DmeCHS1/2*

**Figure 4.**
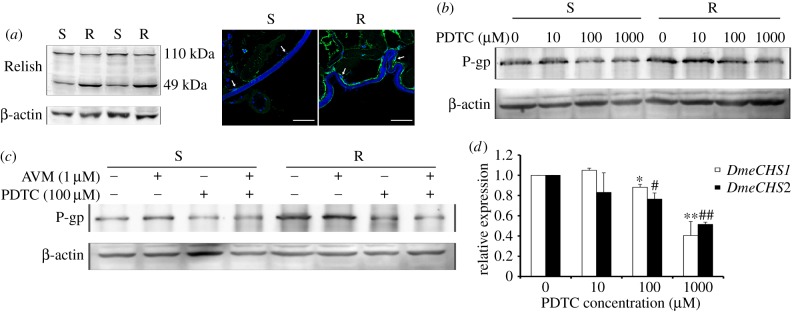
Relish regulates the expression of P-gp and *DmeCHS1/2*. (*a*) The protein level of Relish (cleavage product, 49 kDa: overall length, 110 kDa) in the *Drosophila* larvae was detected by immunofluorescence and western blotting analysis. Blue: nuclei stained by Hoechst33258; green: target proteins (scale bar, 50 µm). (*b,c*) The protein level of P-gp (140 kDa) in the larvae was detected after treatment with different concentrations of Relish activation inhibitor PDTC (*b*), or 100 µM PDTC plus 1 µM avermectin (AVM; *c*), for 48 h. (*d*) The mRNA expression levels of *DmeCHS1* and *DmeCHS2* in R larvae treated by PDTC for 48 h were determined by qPCR analysis with β-actin as the internal control. Data were expressed as mean ± s.e.m., *p < 0.05, **p < 0.01, ^#^*p* < 0.05, ^##^*p* < 0.01, compared with the corresponding control group, *n* = 3.

To determine how the activation of the EGFR/AKT/ERK pathway induces the overexpression of P-gp and *DmeCHS1/2*, we detected the activity of transcription factor Relish (NF-*κ*B) in the larvae. The full-length Relish (110 kDa) is not functional. Upon activation and cleavage, the N-terminal fragment of Relish migrates into the nucleus and serves as a transcriptional factor, whereas the C-terminal fragment (49 kDa) remains in the cytoplasm, and can be used as an indicator for Relish activation [38]. Compared with S larvae, Relish in R larvae was activated (figure 4*a*). To determine whether activation of Relish leads to the overexpression of P-gp and *DmeCHS1/2*, the larvae of the two strains were exposed to different concentrations of Relish activation inhibitor pyrrolidinedithiocarbamic acid (PDTC). PDTC treatment decreased P-gp expression in both of the S and R larvae and decreased *DmeCHS1/2* mRNA levels in R larvae (figure 4*b,d*). Meanwhile, PDTC inhibited P-gp expression in S and R larvae treated with AVM (figure 4*c*).

**Figure 5.**
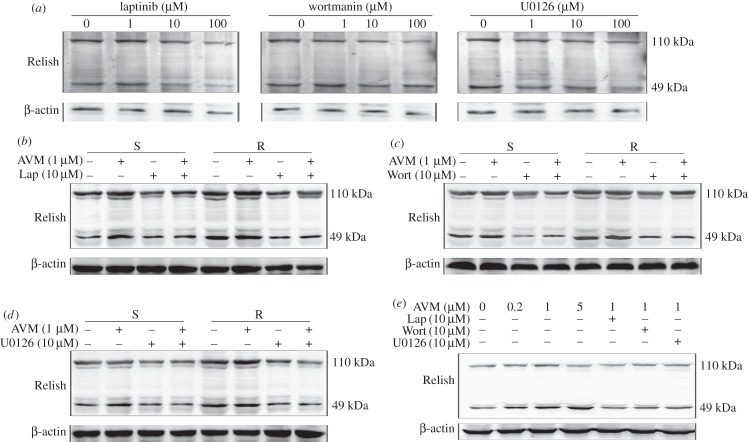
Relish is regulated by EGFR/AKT/ERK signalling pathway. (*a*) The protein level of Relish in avermectin-susceptible (S) larvae treated with different concentrations of lapatinib (Lap), wortmanin (Wort) or U0126 for 48 h. (*b–d*) The protein level of Relish in larvae treated with avermectin (AVM) plus lapatinib (*b*), wortmanin (*c*) or U0126 (*d*) for 48 h. (*e*) The protein level of Relish in S2 cells treated with different concentrations of AVM for 1 h; or pretreated with lapatinib, wortmanin or U0126 for 2 h and then treated with 1 µM AVM for 1 h.

We next investigated the relationship between the EGFR/AKT/ERK pathway and Relish. In the susceptible strain, lapatinib, wortmanin and U0126 inhibited the activation of Relish dose-dependently (figure 5*a*). In addition, lapatinib, wortmanin and U0126 suppressed the expression and activation of Relish in both the S and R larvae, whereas AVM induced the activation of Relish (figure 5*b–d*). Moreover, in S2 cells, AVM induced the activation of Relish dose-dependently and lapatinib, wortmanin and U0126 suppressed the activation of Relish induced by AVM (figure 5*e*). Taken together, our results suggest that AVM and the EGFR/AKT/ERK pathway induces the activation of Relish, which regulates the overexpression of P-gp and *DmeCHS1/2*.

### 2.5. Avermectin directly interacts with epidermal growth factor receptor and activates EGFR/AKT/ERK pathway

**Figure 6.**
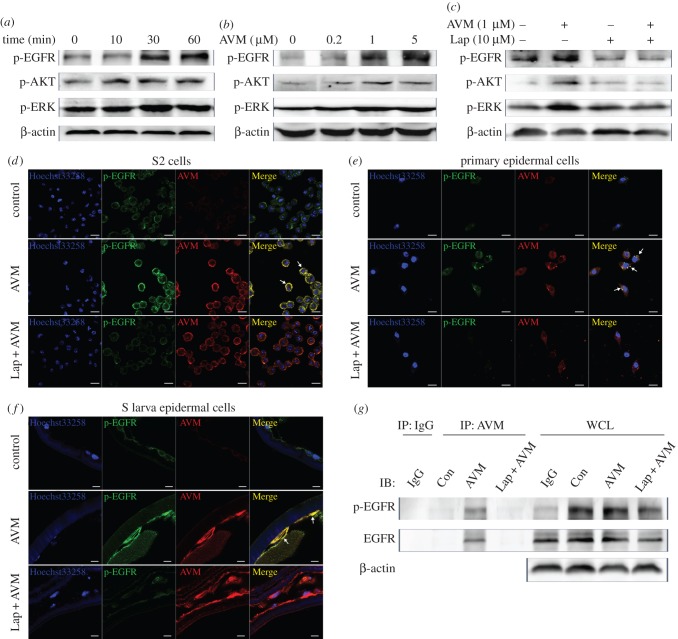
AVM directly interacts with EGFR and activates EGFR/AKT/ERK signalling pathway. (*a–c*) The protein levels of p-EGFR, p-AKT and p-ERK in S2 cells treated with 1 µM avermectin (AVM) for different times (*a*), 0–5 µM AVM for 30 min (*b*) or 1 µM AVM for 30 min plus 10 µM lapatinib (Lap) pretreatment for 120 min (*c*). (*d–f*) The co-localization of AVM with p-EGFR was detected by immunofluorescence. The S2 cells (*d*), primary *Drosophila* epidermal cells (*e*) and avermectin-susceptible larvae (S; *f*) were treated with 1 µM AVM for 30 min (*d,e*) or 1 h (*f*) with or without pretreatment with 10 µM lapatinib for 120 min. Blue: nuclei stained by Hoechst33258; green: target proteins; red: AVM (scale bar, 10 µm). (*g*) The interactions of AVM with EGFR and p-EGFR were determined by co-immunoprecipitation assay in S2 cells. ‘IP’ indicates cell lysates immunoprecipitated with non-specific IgG or anti-AVM antibody. ‘Con’, ‘AVM’, ‘Lap + AVM’ indicates cells treated with vehicle, 1 µM AVM for 30 min and 1 µM AVM for 30 min plus 10 µM lapatinib pretreatment for 120 min, respectively. ‘IgG’ indicates the vehicle-treated cell lysates immunoprecipitated with non-specific IgG. ‘WCL’ indicates the whole cell lysates.

To explore the mechanism of the activation of the EGFR/AKT/ERK pathway in R larvae and determine whether AVM directly or indirectly activates EGFR, we examined the activation of the EGFR/AKT/ERK pathway in S2 cells exposed to AVM. The treatment of AVM dose-dependently and time-dependently activated the EGFR/AKT/ERK pathway within 1 h (figure 6*a,b*). In addition, lapatinib inhibited the activation of the EGFR/AKT/ERK pathway induced by AVM (figure 6*c*). These results suggest that AVM activates the EGFR/AKT/ERK pathway immediately upon contacting the cells, which raises the possibility that AVM directly interacts with the membrane protein EGFR when it contacts cells. To test this possibility, we analysed the co-localization of AVM and EGFR. EGFR and p-EGFR in S2 cells co-localized with AVM after cells were exposed to AVM for 30 min. However, pretreatment with lapatinib prevented p-EGFR from co-localizing with AVM (figure 6*d*). Moreover, in primary epidermal cells from wing imaginal discs and epidermal cells of S larvae, AVM induced the activation of EGFR and co-localized with p-EGFR, and pretreatment with lapatinib blocked these effects (figure 6*e,f*). Furthermore, we found that EGFR and p-EGFR co-immunoprecipitated with AVM in S2 cells and their interactions were disrupted by lapatinib pretreatment (figure 6*g*). In summary, these observations suggest that AVM interacts with EGFR directly, which induces the activation of the EGFR/AKT/ERK pathway.

To further confirm the interaction mode of AVM and EGFR, we docked the AVM B1a onto the three-dimensional structures of the ectodomain of *Drosophila* EGFR. We found that the AVM most likely bound to the domain II of EGFR (electronic supplementary material, figure S4), which is different from EGF [39].

## 3. Discussion

Our study reveals that the EGFR/AKT/ERK/Relish signalling pathway plays pivotal roles in regulating the penetration resistance to AVM in *D. melanogaster* larvae. In addition, AVM interacts with EGFR directly and activates the EGFR/AKT/ERK signalling pathway in *Drosophila* epidermal cells.

This study shows firstly that the decrease of epidermal penetration of AVM is related to the upregulation of *DmeCHS1/2* in AVM-resistant *Drosophila* larvae compared with that in the susceptible larvae. However, an earlier study showed that AVMs can inhibit chitin synthesis in *Mucor miehei* and *Artemia salina* [40]. The difference may be due to the different doses of AVM used in these studies. In addition, the signalling pathways that regulate the chitin synthases in *Drosophila* larvae and *Mucor miehei* may not be the same.

There are notable differences in P-gp expression between *Drosophila* adults and larvae. The upregulation of the AVM efflux-transporter P-gp in BBB was found to confer the resistance to AVM in *Drosophila* adults [30]. Unlike the adults, which have a complete BBB structure, *Drosophila* larvae have an immature one [41–43]. P-gp is mainly expressed in the epidermis and gut of larvae (figure 3*b*), whereas P-gp is mainly expressed in the head of adults [30]. Our results suggest that the efflux-transporter P-gp and chitin synthases can serve as the targets of integrated pest management to resolve the problem of insecticides resistance. However, how much role the two play in the decrease of the penetration of AVM is unknown. Moreover, other mechanisms may be involved in the development of the penetration resistance, which merit further investigation.

Identification of the signalling pathway to regulate the overexpression of P-gp and chitin synthases is crucial for illustrating the mechanism of penetration resistance. Our findings are consistent with earlier reports showing that P-gp expression is regulated by the EGFR signalling pathway [44,45]. The EGFR signalling pathway also plays key roles in the development and homeostasis of epidermal tissues [46]. The previous study showed that the activation of the EGFR/AKT pathway could be detected in AVM-resistant *Drosophila* adults [30]. However, how EGFR/AKT was activated was unknown. Notably, we reveal for the first time that EGFR is indeed a direct target of AVM, and the interaction of AVM with EGFR activates the downstream signalling pathways. The result of our docking analysis suggests that the interaction of AVM and EGFR is different from that of the EGF, which indicates that the mode of binding and activation of AVM to EGFR may be novel. The details of the mechanisms merit further studies. Over-activation of EGFR and its downstream signalling pathways is prominent in drug-resistant tumour tissues [47,48]. Thus, the interaction of drug and EGFR may be responsible for the over-activation of EGFR signalling pathways in cancers.

In this study, we discovered that the transcription factor Relish (NF-*κ*B) was activated by EGFR and induced the overexpression of chitin synthases and P-gp. Relish (NF-*κ*B) can be activated in a wound-healing model of *Drosophila* larvae [49]. In the non-activated state, Relish binds with I-κB, which inhibits the activation of Relish [50]. The phosphorylation and degradation of I-*κ*B can induce the cleavage of Relish and the nuclear translocation of the N-terminal fragment of Relish [51]. AKT and ERK can regulate the phosphorylation and degradation of I-κB by phosphorylating relevant kinases [52,53]. NF-κB can regulate P-gp expression by directly inducing the MDR1 gene transcription [54]. Thus, the EGFR/AKT/ERK/Relish pathway may also serve as a target for resolving the insecticide resistance. Note that in our earlier study, we found that in S2 cells, Ca^2+^ signalling activated Relish and the overexpression of P-gp upon AVM treatment [55]. It is likely that the Ca^2+^ signalling pathway has crosstalk with the EGFR signalling pathway, which merits further investigation.

**Figure 7.**
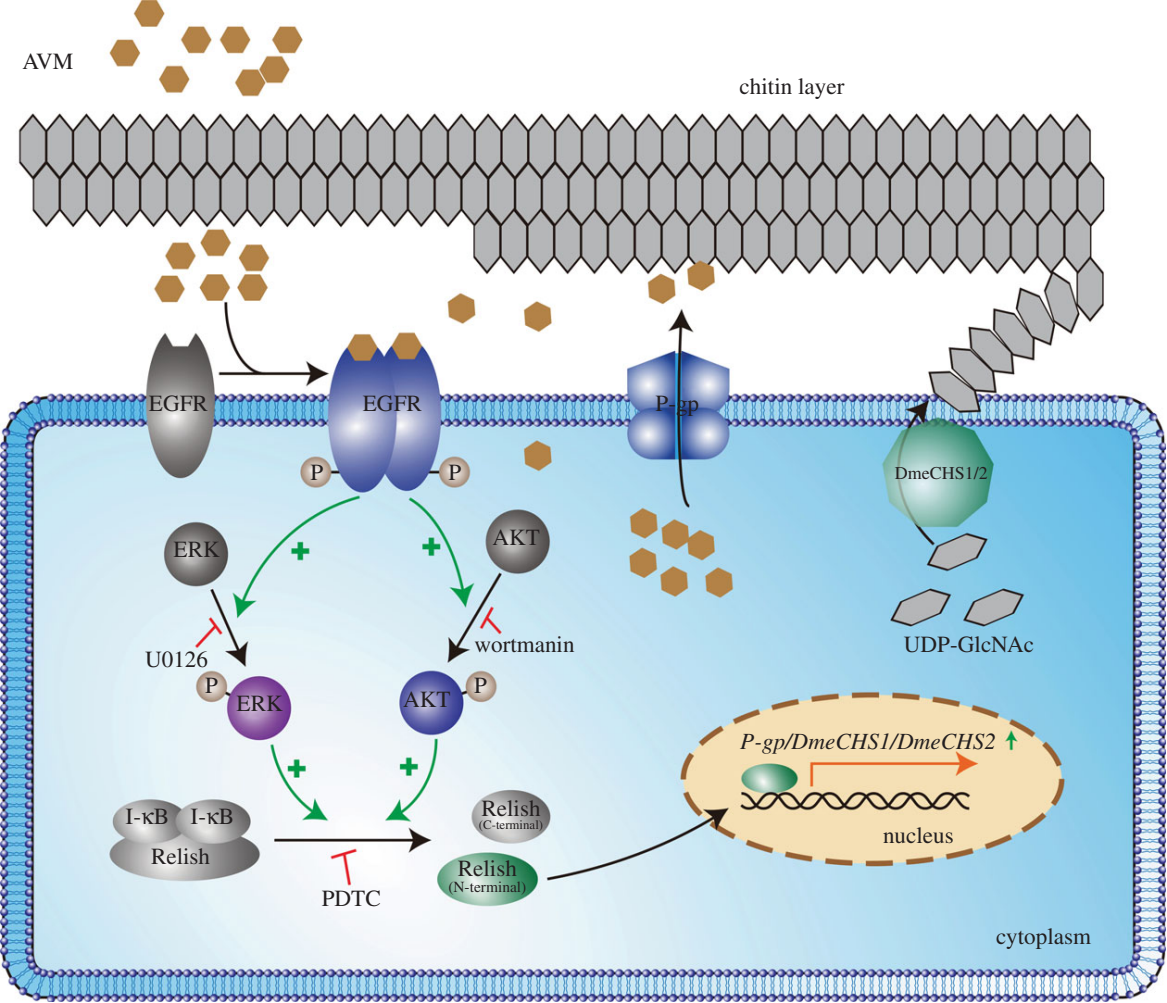
Schematic of the mechanism of avermectin-induced penetration resistance.

In summary, the epidermal penetration of AVM drastically reduces in AVM-resistant *Drosophila* larvae, compared with the sensitive larvae, owing to the elevated expression of the efflux transporter P-gp and the thickened chitin layer in the epidermis. Furthermore, AVM directly interacts with EGFR and activates the EGFR/AKT/ERK/Relish pathway to induce the overexpression of P-gp and chitin synthases in *Drosophila* epidermal cells. Altogether, these findings provide new insights into the mechanisms underlying insecticide resistance and have important implications for drug resistance biology (figure 7).

## 4. Material and methods

### 4.1. Chemicals and reagents

AVM (containing 93% avermectin B1a and 7% avermectin B1b) was obtained from the ZND Bio-technology Co., Ltd. (Beijing, China), and the anti-AVM antibody was a gift from Professor Shen in CAU (Beijing, China). The procedures for preparing the anti-AVM antibody were as follows. First, the structure of AVM was modified by succinylation. Then, the 4″-*O*-succinyl-AVM was conjugated to bovine serum albumin (BSA). This immunizing antigen was then injected into rabbits, and the polyclonal antibody was acquired [56].

Lapatinib (EGFR phosphorylation inhibitor) was purchased from Selleck (Houston, TX). Wortmanin (phosphoinositide-3-kinase (PI3K) inhibitor) and U0126 (mitogen-activated protein kinase kinase (MEK) inhibitor) were purchased from Kinasechem (UK). The Relish inhibitor pyrrolidinedithiocarbamic acid (PDTC) was purchased from Sigma-Aldrich (St Louis, MO). The mouse monoclonal anti-P-gp antibody (C219) was purchased from Calbiochem (Darmstadt, Germany). Anti-p-AKT antibody, anti-p-ERK antibody, anti-AKT antibody and anti-ERK antibody were purchased from Cell Signaling Technology (Boston, MA). Anti-EGFR antibody was purchased from Santa Cruz Biotechnology (Dallas, TX). Anti-p-Tyr antibody was purchased from BD Biosciences (Franklin Lakes, NJ). Anti-Relish antibody was purchased from the Developmental Studies Hybridoma Bank (University of Iowa, Iowa City, IA).

### 4.2. Selection of avermectin-resistant fly and culture of fly

The *w*^1118^ strain *D. melanogaster* was used as the AVM-susceptible strain (S). To obtain the AVM-resistant strain (R), we exposed susceptible *D. melanogaster* to AVM in standard fly medium prepared according to the method used previously [30]. AVM concentration was raised stepwise from 20 nM to 1.5 µM in 60 months after 100 generations. The resistant strain can survive and reproduce in the medium containing 1.5 µM AVM. The resistance ratio of larvae (see below) was about 50. The third-instar larvae of the same size were used in the experiment. All flies were cultured at 25°C and 70% humidity.

### 4.3. Topical drug treatment

Different concentrations of AVM were dissolved in acetone. The third-instar larvae of the same size were chosen. The adults of the same sizes were also used. A drop of AVM solution (0.25 µl) was applied with a hand microapplicator (Burkard, England) to the dorsum of the larvae and the abdomen of adults lightly anaesthetized on ice. Control larvae and adults were treated in the same way with acetone. Larvae and adults were fed with fresh medium and kept at 25°C and 70% humidity. For each assay, 50 third-instar larvae and adults were treated, and the whole assay was repeated three times.

### 4.4. Toxicity detection of fly larvae and adults

The LD_50_ of *Drosophila* adults and larvae to AVM was determined by using the topical drug treatment. Each treatment group contains thirty larvae or adults of the same size. Each *Drosophila* larva or adult was spotted with 0.25 µl different concentrations of AVM and cultured in the standard medium for 24 h. The AVM was dissolved in acetone. The LD_50_ was calculated according to the mortality of *Drosophila* at different concentrations of AVM. The resistance ratio was the ratio of the LD_50_ of the R strain and the LD_50_ of the S strain.

### 4.5. Cell culture

S2 cell line was a gift from Professor Li (Institute of Zoology, Chinese Academy of Sciences, Beijing, China). S2 cells were cultured in Hyclone insect medium (Thermo, Waltham, MA) at 26°C. The primary *Drosophila* wing epithelial cells were obtained by culturing the imaginal discs of larvae according to the previous report [57].

### 4.6. Chemical treatments of larvae

*Drosophila* larvae were treated with various concentrations of AVM, lapatinib, wortmanin, U0126 and PDTC. The chemicals were dissolved in DMSO to make stock solution and then diluted in double distilled H_2_O (ddH_2_O) to make working solutions, which were mixed with standard fly medium (1 : 1, v : v). *Drosophila* larvae, susceptible or resistant to AVM, were transferred from standard medium into chemical-containing medium. After 48 h treatment, the larvae were collected and washed in ddH_2_O, dried by using absorbent paper and weighed. The larvae samples were then prepared for western blotting analysis or HPLC analysis.

### 4.7. Western blotting analysis

Cells or larvae were lysed in buffer containing 50 mM Tris, pH 7.5, 150 mM NaCl, 1% Triton X-100, 10% glycerol, 1 mM EDTA, 1% sodium deoxycholate, 1 mM PMSF and 1% protease inhibitors. Lysates were centrifuged at 12 000 r.p.m., 4°C for 15 min. Supernatants were boiled with the loading buffer for 5 min. The protein samples were electrophoresed in SDS–polyacrylamide gels and then transferred onto Millipore PVDF membranes (Darmstadt, Germany). The membranes were blocked in PBST buffer containing 5% fat-free milk (w/v) for 1 h at room temperature (RT), incubated with the corresponding antibody at 4°C overnight, then incubated with the secondary antibody conjugated to horseradish peroxidase for 2 h at RT. Membranes were stained with standard ECL reagents purchased from ComWin Biotech (Beijing, China) and then photographed by DNR MicroChemi4.2 system (Bio-Imaging Systems Ltd, Israel).

### 4.8. Immunofluorescence analysis

The larvae tissue frozen sections and S2 cells were fixed in 4% paraformaldehyde for 10 min at RT, blocked in PBST containing 3% BSA for 1 h at 37°C, incubated with the first antibody at 37°C for 1 h, then incubated with the appropriate fluorescent probe-labelled secondary antibody for 1 h at 37°C. The nuclei were stained with Hoechst33258 for 10 min at RT. All images were acquired using a Carl Zeiss LSM710 laser scanning confocal microscope (Oberkochen, Germany).

### 4.9. Quantitative PCR analysis

The relative mRNA level of *DmeCHS1* and *DmeCHS2* in *Drosophila* was determined by quantitative PCR using a TaKaRa SYBR *Premix Ex* Taq™ (Tli RNaseH Plus) PCR kit (Dalian, China). Each group of treated larvae weighing 100 mg was homogenized with 1 ml Trizol reagent (Invitrogen, Carlsbad, CA) in a glass homogenizer on ice. Then, the mixture was placed at RT for 10 min. 200 µl CHCl_3_ was added, and the tubes were vortexed for 1 min and centrifuged at 12 000 r.p.m. for 15 min at 4°C. The supernatant was transferred into a new centrifuge tube, mixed with 500 µl isopropanol, vortexed for 1 min and centrifuged at 12 000 r.p.m. for 10 min at 4°C. The precipitant was washed with 70% ethanol twice, dried and dissolved in 0.1% DEPC H_2_O. The total RNA concentration was measured using a Biophotometer plus (Eppendorf, Hamburg, Germany). Total RNA (1 µg) was reverse-transcribed into cDNA by using a M-MuLV reverse transcriptase assay kit (Fermentas, Ontario, Canada). Quantitative PCR assay was carried out with a MX3000P real-time PCR thermocycler (Axygen, California, USA). The PCR primers used are listed in the electronic supplementary material, table S1.

### 4.10. Transmission electron microscopy

The larvae were dissected in PBS on ice to obtain the larva body wall. The tissues were fixed in the fixative solution containing 4% paraformaldehyde and 2.5% glutaraldehyde for 24 h. Then, the tissues were fixed in 1% OsO_4_, dehydrated stepwise in ethanol solutions and embedded in resin. After 48 h polymerization at 60°C, the tissues were sliced at 200 or 100 nm thickness. The 200 nm slices were stained by 1% toluidine blue (dissolved in 1% Borax solution) for 1 min and observed using an optical microscope (Olympus, Tokyo, Japan). The 100 nm slices were plated on the copper network, stained with uranyl acetate for 10 min in the dark, washed with PBS three times, stained with lead citrate for 30 min and washed with PBS. The images were acquired using a JEOL-1010 transmission electron microscope (JEOL Ltd., Japan).

### 4.11. HPLC analysis of avermectin permeability

After 1 h starvation in ddH_2_O, each larva was spotted by 0.25 µl of 10 mM AVM solution and cultured in common corn medium for 2 h. The larvae bodies were washed with acetonitrile three times to obtain the sample for AVM on the surface of the larvae body. Then, the larvae were homogenized with acetonitrile on ice and centrifuged at 12 000 r.p.m. for 15 min at 4°C. The supernatant was used as the sample for AVM that has penetrated into the larvae body.

All the samples were filtered with 0.22 µm filters, blow-dried with nitrogen and then dissolved in 60 µl acetonitrile. Then the samples and the internalized samples were treated with the trifluoroacetic anhydride (TFAA)–*N*-methyl imidazole (NMIM)–acetonitrile (ACN) method. First, 10 µl of a mixture of 1-methylimidazole and acetonitrile (1/1, v/v) was added to the sample vials. Then, 20 µl of a mixture of TFAA and acetonitrile (1/2, v/v) was added to the sample vials. The sample vials were vortexed for 30 s and derivatized for 15 min in the dark. After the derivatization, 30 µl methanol was added to the sample vials, and then vortexed for 1 min. The AVM residues were detected with the Agilent 1100 series HPLC system (California, USA). The amount of AVM residue was calculated according to the standard curve. AVM permeability was calculated as the ratio of the amount of internalized AVM and the sum of the amount of internalized AVM and AVM on the surface of the larvae body.

### 4.12. Docking analysis

The Docking analysis of AVM B1a and *Drosophila* EGFR was carried on the AutoDock. The molecule files were modified for docking by using ADT software [58]. The PDB file of AVM B1a was constructed by ChemBio3D. The code of PDB file of the *Drosophila* EGFR ectodomain is 3I2T. The AVM B1a was chosen as flexible ligand, and EGFR was chosen as rigid receptor. Ten models with the lower binding energy were shown in ADT. The docking model with the lowest binding energy was chosen as a result.

### 4.13. Statistical analysis

All experiments were repeated at least three times. Data were expressed as the means ± standard error. For statistical analysis, Student's *t*-tests were used to compare paired data and a one-way analysis of variance followed by Dunnett's test was used for multiple comparisons. Values of *p* < 0.05 were considered significant; values of *p* < 0.01 were considered extremely significant.

## Supplementary Material

Supplementary figures
