# Supplementary material for: Direct interaction of avermectin with epidermal growth factor receptor mediates the penetration resistance in Drosophila larvae
Source: Open Biol. 2016 Apr 13;6(4):150231. doi: 10.1098/rsob.150231 (PMC4852453; doi:10.1098/rsob.150231)
Supplement: Supplementary figures [file rsob150231supp1.pdf]

Supplementary materials

Title of the MS: Direct interaction of avermectin to EGFR mediates the penetration resistance in *Drosophila* larvae

Authors: Chen L-P, et al.

Supplementary figures

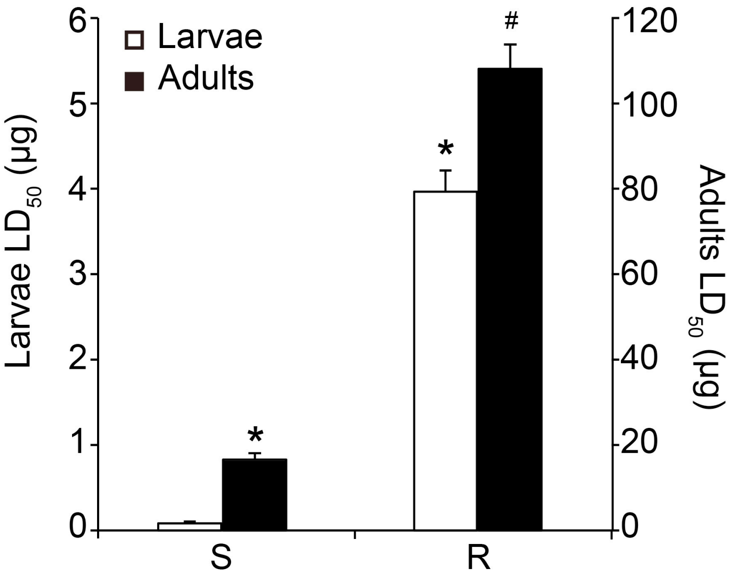

Figure S1. The LD_{50} of the *Drosophila* larvae and adults exposed to avermectin. The larvae and adults were spotted with 0.25 μl avermectin and/or acetone and cultured in standard medium for 24 h. The value of LD_{50} was calculated according to the mortality of larvae and adults exposed to different doses of avermectin (AVM) with *Probit* regression analysis. Data were expressed as mean ± SEM, $^{*}P < 0.01$, $^{\#}P < 0.01$, compared to the corresponding S control group. n=3. S and R are short for AVM-susceptible strain and AVM-resistant strain, respectively.

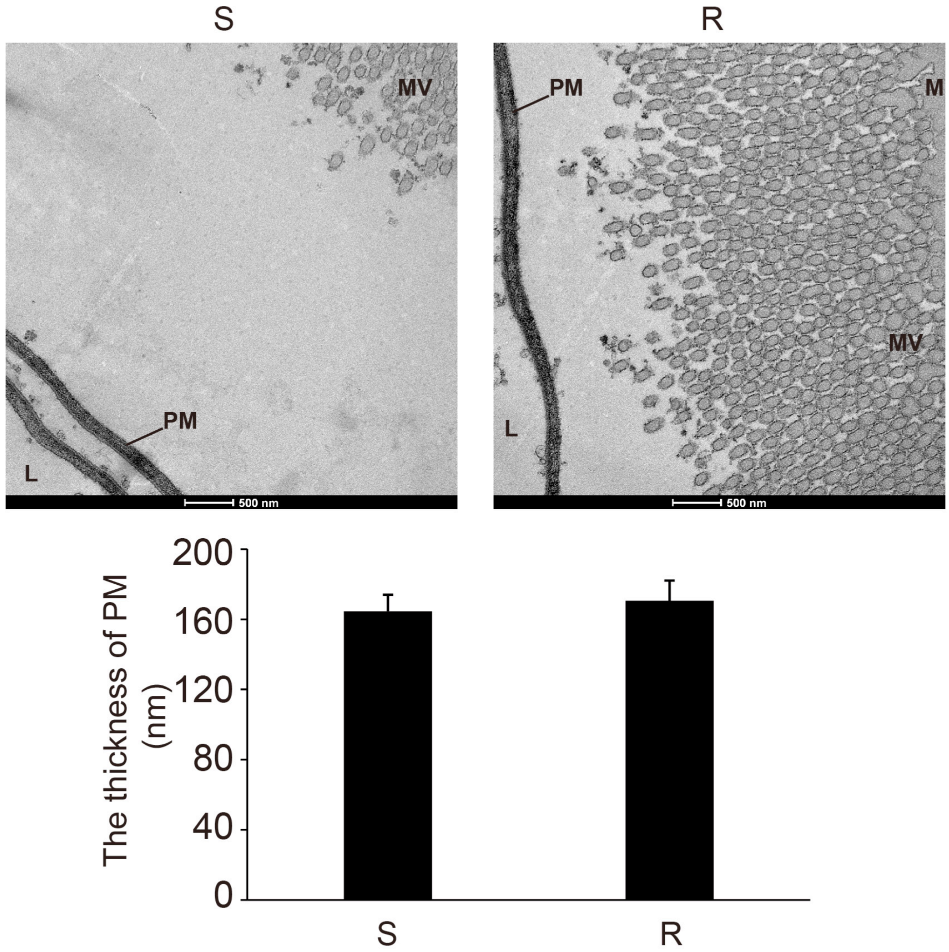

Figure S2. The peritrophic membranes in the midgut of the *Drosophila* larvae. (**A**) Section through the peritrophic membranes (PM) surrounded by the microvillar brush border (MV) and luminal (L). (**B**) The thickness of PM determined by electronic microscope. S and R are short for avermectin-susceptible strain and avermectin-resistant strain, respectively.

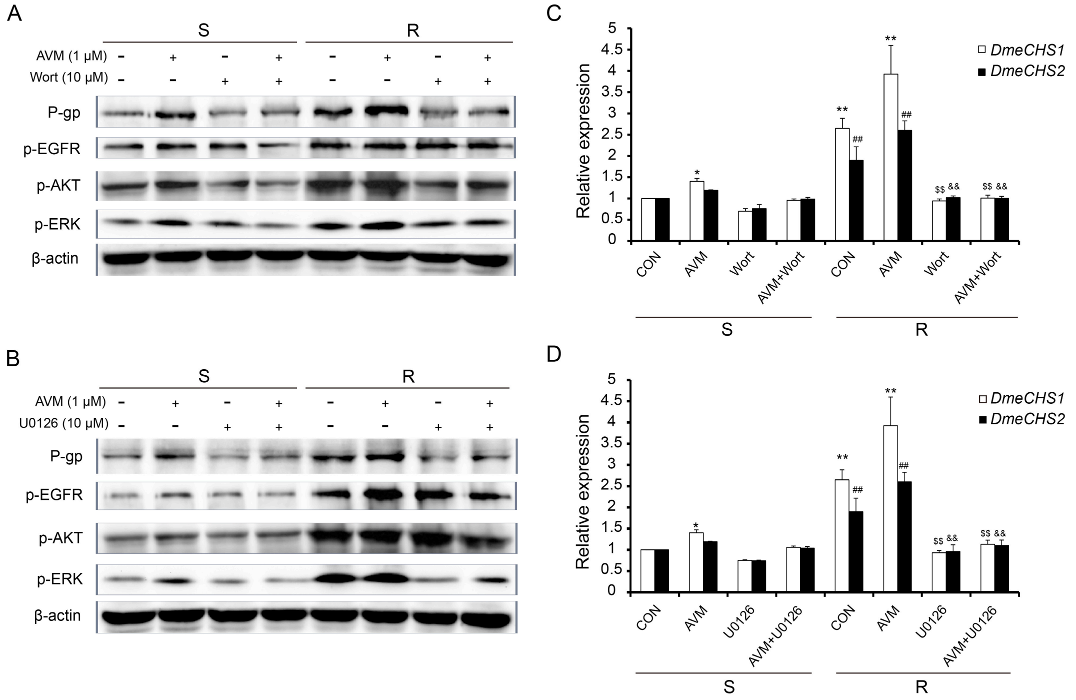

Figure S3. Expression of P-gp and DmeCHS1/2 is regulated by EGFR/AKT/ERK signaling pathway. The S and R *Drosophila* larvae were treated with avermectin (AVM), wortmanin (Wort) or U0126 for 48 h. (**A-B**) The protein levels of P-gp, p-EGFR, p-AKT, and p-ERK in larvae treated with AVM and wortmanin (**A**), or U0126 (**B**) were determined by Western blotting analysis. (**C-D**) The mRNA levels of *DmeCHS2* and *DmeCHS1* in larvae treated with AVM and wortmanin (**C**), or U0126 (**D**) were determined by qPCR analysis with β-actin as the internal control. Data were expressed as mean ± SEM, $^{*}P< 0.05$, $^{**}P< 0.01$, $^{\#}P< 0.05$, $^{\#\#}P< 0.01$, compared to the corresponding S control group; $^{\$}P< 0.05$, $^{\$\$}P< 0.01$, $^{\&}P< 0.05$, $^{\&\&}P< 0.01$, compared to the corresponding R control group; n= 3. CON, S, R, and Wort are short for control, AVM-susceptible strain, AVM-resistant strain, and wortmanin, respectively.

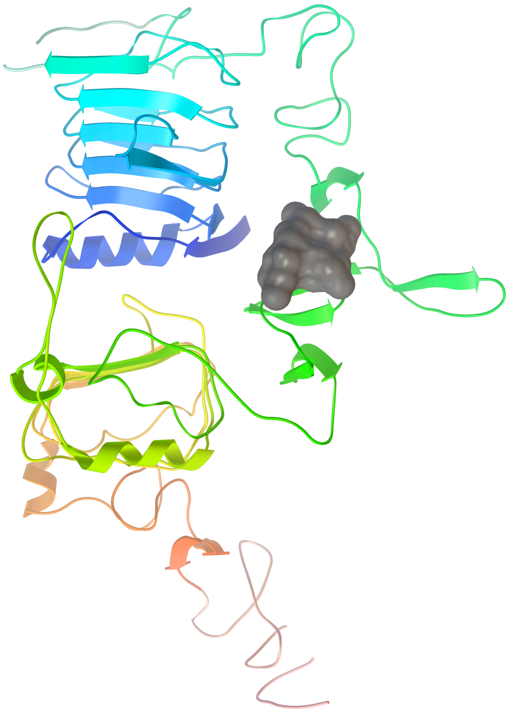

Figure S4. Docking model of the avermectin binding to EGFR. Ribbon representation of the ectodomain of *Drosophila* EGFR, with domain I colored blue, domain II green, domain III yellow, and domain IV red. Space-filling representation of avermectin B1a is colored gray.

Supplementary table

Table S1 List of primers used in the study

**Primers for production of dsRNA**	
EGFRATPbi-F	GAATTAATACGACTCACTATAGGGAGAGGCTCTAAGGCTCTGCTCAACT
EGFRATPbi-R	GAATTAATACGACTCACTATAGGGAGATTGGGTATCTCATCCGTGC
Mdr49ATPbi-F	GAATTAATACGACTCACTATAGGGAGAAGGGCATACGCTTCCGTTA
Mdr49ATPbi-R	GAATTAATACGACTCACTATAGGGAGACCAGTTCGCAGTAGAGACCA
Mdr50ATPbi-F	GAATTAATACGACTCACTATAGGGAGATGGCTCTTGTTGGTCCAT
Mdr50ATPbi-R	GAATTAATACGACTCACTATAGGGAGACTCTTCCTCATCTTCATCCTTG
Mdr65ATPbi-F	GAATTAATACGACTCACTATAGGGAGAGGTTCTTCTGGCTGTGGAA
Mdr65ATPbi-R	GAATTAATACGACTCACTATAGGGAGACGTCTCAAAGGATTTCTCAAAC
**Primers for Fluorescence Quantitative PCR**	
DmeCHS2-F	GGAGGAGACTACGATAGCGGTGAC
DmeCHS2-R	GCGAAGACAAGGAGGTAGGTGC
DmeCHS1-F	AACAAAGACTTGGGTCGGGACA
DmeCHS1-R	CCATCAGCCAGACGAAGAGGA
β-actin-F	CGTCCGTGACATCAAGGAGAAGC
β-actin-R	CCAAGAACGAGGGCTGGAACA
**Primers for Reverse Transcription PCR**	
DmeCHS2-F	GCAGCCCCACAATGACAGG
DmeCHS2-R	CCTCATCCAAGCGGACAAT
DmeCHS1-F	GCCAACGCACCATACAAGAGACG
DmeCHS1-R	CTGGGGCAGAACCACGAACATCA
β-actin-F	ACCGAGGCCCCGCTGAACCCCAAGG
β-actin-R	AGAACGAGGGCTGGAACA
